# The Social Life of Class Clowns: Class Clown Behavior Is Associated With More Friends, but Also More Aggressive Behavior in the Classroom

**DOI:** 10.3389/fpsyg.2019.00604

**Published:** 2019-04-26

**Authors:** Lisa Wagner

**Affiliations:** Personality and Assessment, Department of Psychology, University of Zurich, Zurich, Switzerland

**Keywords:** class clown, humor, school, adolescence, peer relationships, peer acceptance, likeability, disruptive behavior

## Abstract

A dimensional rather than a typological approach to studying class clown behavior was recently proposed (Ruch et al., 2014). In the present study, four dimensions of class clown behavior (class clown role, comic talent, disruptive rule-breaker, and subversive joker) were used to investigate the associations between class clown behavior and indicators of social status and social functioning in the classroom in a sample of *N* = 300 students attending grades 6 to 9 (mean age: 13.09 years, 47.7% male). Participants and their teachers completed measures of class clown behavior, and peer nominations of peer acceptance, mutual friends as well as social behavior in the classroom (popular-leadership, aggressive-disruptive, sensitive-isolated, and prosocial behaviors) were collected. The results showed that overall, class clown behavior was positively related to peer acceptance, the number of mutual friends in the classroom and peer-perceived social status. Overall, it was also positively related to peer-rated popular-leadership and aggressive-disruptive behaviors, as well as negatively related to prosocial behaviors. When considering the four dimensions of class clown behavior, comic talent was particularly relevant for the relationship with social status and with popular-leadership behaviors, but also with aggressive-disruptive behaviors. Aggressive-disruptive behaviors were also particularly related to the class clown dimension disruptive rule-breaker. The results underline the significance of class clown behavior for the social status and functioning of students and may help further understand the phenomenon in its multidimensional nature.

## Introduction

Relationships with peers impact well-being throughout the entire life span. However, the influence that peers exert on many areas of life seems to be most pervasive in early adolescence (see Parker et al., 2006). The impact of peer relationships spans from the development of cognitive and social skills, to maladaptive functioning and physical and psychological well-being (e.g., Hartup and Stevens, 1999; Parker et al., 2006; Rubin et al., 2015). Of particular importance for child adolescent development are peer relationships and social functioning in the classroom (e.g., Berndt and Ladd, 1989). In terms of how to assess social functioning in the classroom, peer nomination procedures such as the Revised Class Play (Masten et al., 1985), have demonstrated their ability to predict important life outcomes, such as academic and job success, social and romantic competence as well as internalizing and externalizing symptoms, for time spans of up to 10 years (Gest et al., 2006). Four areas of social behavior are typically distinguished (e.g., Realmuto et al., 1997; Zeller et al., 2003), two of which are adaptive (popular-leadership and prosocial behavior) and two of which are maladaptive (sensitive-isolated and aggressive-disruptive).

Next to social behavior in the classroom as perceived by the classmates, also the social status, that is peer acceptance and the number of mutual friends in the classroom, is an important indicator of positive peer relationships in adolescence, that has demonstrated its relevance for many important life outcomes. Peer acceptance is a unilateral construct that describes being liked by one’s classmates, whereas mutual friends are defined by a bilateral understanding, i.e., friendship nominations by both friends (Rubin et al., 2006; Waldrip et al., 2008; Bagwell and Schmidt, 2011). While many studies have focused on the impact of having versus not having a mutual friend, research also shows that it matters how many mutual friends an adolescent has. For instance, Gest et al. (2001) found the number of friends to uniquely predict prosocial behavior. Students who are highly accepted, that is well-liked, by their peers have been found to show a range of highly desirable characteristics, for example behaving appropriately, communicating well, and being perceived as helpful, cooperative, and good leaders by other students (Rubin et al., 2006). Peer acceptance was also found to be related to academic and athletic accomplishments (e.g., Asher and McDonald, 2009). In addition, being well-accepted by peers and having many friends has also been associated with individual differences in sense of humor (e.g., McGhee, 1989; Wanzer et al., 1996; Gest et al., 2001). In conclusion, both peer acceptance and the number of friends a student has in the class are indicators of social status that go along with a number of important outcomes.

Humor has been identified as a strength of character, and humans tend to find the expression of their signature (i.e., most characteristic) character strengths fulfilling (Peterson and Seligman, 2004). Individuals with the signature strength of humor tend to express humor in their behavior across different contexts and might thus earn a reputation for their humor in their social networks which might result in others using type nouns (like joker, wit, buffoon, or mocking bird) to refer to them (Craik and Ware, 2007). Often people using humor play a function in the institution they are in, such as the “organizational fool” (Kets de Vries, 1990) and the “class clown” (e.g., Damico and Purkey, 1978). Humor is a way of highlighting signs of hubris in leaders, of addressing taboo topics, and of relaxing strained situations. Thus, the institutional fool may satirize leaders and followers. As the truth is spoken in a fun manner, it can trespass on otherwise forbidden territory. Thus, the organizational fool can create a corrective force against the leadership in institutions and be a mediator between leader and followers.

There are comparable circumstances in the classroom where teachers typically socialize children into school culture. The teacher mostly decides who will speak, when, and about what, and also decides what he or she can conceive from the students. Oppositional students – and among these, students considered class clowns – might negotiate power in their classroom communities, and try to resist the set order during classroom lessons (McLaren, 1985; Radigan, 2001; Norrick and Klein, 2008). A class clown may become the opponent of the teacher; poke fun at the teacher’s words and action and undermine his authority, in front of the teacher or behind their backs. From the teachers’ perspective, students described as class clowns are mostly viewed as difficult students that require being disciplined (see e.g., Cohen and Fish, 1993; Hobday-Kusch and McVittie, 2002). Analyzing accounts of humorous situations in the classroom initiated by students toward teachers, Meeus and Mahieu (2009) found that while testing out, rebellion, and misbehavior were common motives identified, also positive motives, such as humor as atmosphere maker, played a role in these accounts.

The first significant study of class clowns (Damico and Purkey, 1976, 1978) identified 96 mostly male class clowns in a sample of 3,500 eighth graders. Compared to a control sample, class clowns were seen by teachers as significantly higher on asserting behaviors, attention seeking, unruliness, leadership, and cheerfulness, but lower in accomplishing, which was defined as “behaviors leading to successful completion of academic assignments” (p. 393). Relative to other students, the class clowns self-reported seeing school authorities, such as teachers or the principal, less positively, but there was no significant difference in class clowns’ attitudes toward classmates, the school in general, and the self. In this study by Damico and Purkey (1978), only students that received 10 or more nominations by their peers were considered a class clown and those that received 25 or more nominations were “super class clowns.” However, it must be acknowledged that there was a large variation in the frequency of nominations and more or less arbitrary cut-off points were used to identify the group of class clowns.

Going beyond previous conceptualizations of class clowns as a distinct “type” or categorical concept, Ruch et al. (2014) suggested an alternative approach to study class clown behavior, which is based on a variable-centered or dimensional view. That is, they assume a general dimension of class clown behavior, but also different related facets that can be used to describe class clown behavior further. These can be assessed using the Class Clown Behavior Survey (CCBS; Platt, 2012). The hierarchical model proposes a general factor of class clown behavior (as measured by the total score of the CCBS) as well as four lower-order dimensions of class clown behavior, which are positively correlated with class clown behavior. The first dimension, *class clown role*, consists of being labeled as the class clown by oneself and others (sample item of the CCBS: “My classmates would call me a class clown”). The second dimension, *comic talent*, describes being quick-witted, liking to entertain others with funny things, and spreading a good mood. It describes behavior that is not necessarily directed against the teacher or questioning the rules at school (sample item: “During class it does not take long until something funny comes into my mind that I can share with the person next to me”). The third dimension, *disruptive rule-breaker*, is characterized by poking fun at the teachers, not taking school rules seriously, and directly challenging the authority (sample item: “Some rules in class I find stupid and I laugh at them”). Similarly, in the fourth dimension, *subversive joker*, the class clown behavior is also aimed at undermining the teachers’ authority or directed against classmates. However, it is done behind the teacher’s back instead of in a direct confrontation (sample item: “When my teacher turns away, I invent jokes that I write on paper to show it to my classmates”).

Ruch et al. (2014) found that all four dimensions went along with higher scores in the character strength of humor, but lower scores in strengths related to restraint, such as self-regulation or prudence. In addition, the dimension *comic talent* went along with higher scores in the strengths of leadership, perspective, zest, social intelligence, bravery, hope, love, and creativity. Platt et al. (2016) added to these results by demonstrating that class clown behavior was associated with lower school satisfaction and lower GPA. When considering the four dimensions, it was only the dimension *disruptive rule-breaker* that related to lower school satisfaction, and only the dimensions *disruptive rule-breaker* and *class clown role* that were negatively related to GPA. On the other hand, the two dimensions *class clown role* and *comic talent* were associated with the experience of more positive emotions in the classroom. Also, while those with high scores on class clown behavior described the relationship with teachers as considerably worse than those with lower scores, the relationships with classmates did not seem to be affected negatively. The dimension *comic talent* had a small but positive correlation with positive relationships with classmates, though it failed to reach statistical significance. Overall, the results of these two studies suggest that different dimensions of class clown behavior can be distinguished, that class clown behavior has both upsides and downsides, and that looking beyond the question of whether or not someone is labeled as “class clown” provides the possibility to gain a deeper insight into the correlates of class clown behavior.

There is some evidence of a relationship between class clown behavior and social status. Damico and Purkey (1978) concluded that their adolescent class clowns were found to have many behaviors and personal assessments in common with adult wits, and among the list of attributes (e.g., being male, leaders, active, independent, creative, and having positive self-perceptions), class clowns are also described as more popular. Likewise, Suitor et al. (2004) report that for male adolescents in private schools, class clowning is a successful route to gain prestige (more so than clothes or car ownership). A recent study by Barnett (2018) looked at the consequences of younger children’s playfulness in the classroom. The study found peer-rated social status to be unrelated with self-ratings of being a class clown (which had a rather low mean and variance) and positively related with peer-ratings of being a class clown. This study also underlined the relevance of sex differences: Although the relationships were present in boys and girls in the peer ratings, teachers had a stronger tendency to designate boys as “class clowns” than girls and teacher-rated class clown status was positively related to social status for girls, and negatively related to social status for boys.

## The Present Study

The present study investigates the social functioning and social status of students showing class clown behavior. For this purpose, self- and teacher ratings of class clown behavior were considered as they reflect two important perspectives that were previously found to converge moderately at best (see e.g., Barnett, 2018). More concretely, the present study deals with the relationships between self- and teacher-reported class clown behavior and (a) peer acceptance, number of mutual friends in the classroom, and peer-perceived social status as well as (b) peer-perceived classroom behavior describing social functioning. It adds to the existing research on class clown behavior by being the first one to investigate the dimensional approach to class clown behavior in relation to various aspects of social relationships in the classroom, by comparing the contribution of both a “type” approach (considered a class clown or not) and a dimensional approach to predicted social status and social functioning in the classroom, and by also considering teacher ratings on the dimensions of class clown behavior and considering their convergence with self-ratings.

Based on the associations between social status and class clown behavior described previously (e.g., Damico and Purkey, 1978). It was expected that class clown behavior would be positively related to peer acceptance as well as to number of friends and peer-perceived social status. In particular the dimension *comic talent* was expected to show the strongest links with these three variables as previous studies on the dimensional approach (Ruch et al., 2014; Platt et al., 2016) hinted at its relevance for positive (peer) relationships. With regards to social functioning, it was expected that the behavioral dimension *popular-leadership* would positively relate to class clown behavior (again, based on the previously established associations between class clowning and both popularity and leadership behavior, see e.g., Damico and Purkey, 1978; Masten, 1986), and again in particular to the dimension of *comic talent*. Based on previous research demonstrating a negative association between teacher-rated social behavior in the classroom and class clown behavior (Platt et al., 2016), it was expected that the behavioral dimension *prosocial* would be negatively related to class clown behavior. Prosocial behavior as described in the RCP is consistent with other-directed strengths and strengths of restraint. In these areas, students displaying class clown behavior, in particular in the *disruptive rule-breaker* dimension, tended to score lower (Ruch et al., 2014), which supports the present hypothesis. Finally, a positive relationship between class clown behavior and the behavioral dimension of *aggressive-disruptive* was expected. In particular the class clown behavior dimension of *disruptive rule-breaker* was expected to be related to aggressive-disruptive classroom behavior as this dimension most directly contains disruptive behaviors and it showed strong negative relationships with teacher-rated positive classroom behavior (Platt et al., 2016).

## Materials and Methods

### Participants

A sample of 300 students (47.7% male) aged on average 13.09 years (*SD* = 1.12; ranging from 11 to 17 years) participated in the study. They attended the sixth (28.7%), seventh (38.7%), eighth (21.3%), or ninth (11.3%) grade in nine different schools in German-speaking Switzerland and Liechtenstein. The number of participating students in each classroom ranged between 11 and 26, with an average of 19.40 (*SD* = 3.44). All students in one classroom typically spent (almost) the entire school day together; as a consequence, they can be assumed to be highly familiar with their peers’ behavior in the classroom and during different lessons.

Classroom teachers (*N* = 17) completed the teacher ratings for students in each classroom. These classroom teachers (41.2% male, 11.8% missing information) were on average 39.40 years old (*SD* = 11.31; ranging from 26 to 64 years) and had on average 13.07 years of teaching experience (*SD* = 10.39; ranging from 3 to 40 years). They knew the students they were rating for on average 3.67 semesters (*SD* = 1.29) and were teaching them regularly, for on average 14.67 lessons (*SD* = 8.72) a week.

### Instruments

In order to address the research questions, self-reports and teacher ratings on class clown behavior and peer ratings of classroom behavior were collected. In addition, a nomination procedure to assess peer acceptance and the number of mutual friends was used.

#### Class Clown Behavior

The *Class Clown Behavior Survey* (CCBS; Platt, 2012) is a self-report instrument assessing different class clown behaviors using 18 items with a 6-point answer format (ranging from 1 = *totally disagree* to 6 = *totally agree*). The survey measures class clown behaviors both as total score and in the form of four subscales of *class clown role*, *comic talent*, *disruptive rule-breaker*, and *subversive joker* (encompassing 4 or 5 items each; see Ruch et al., 2014). In the present sample, the total score yielded an internal consistency of α = 0.94 and the subscales yielded coefficients of α = 0.90, α = 0.87, α = 0.87, and α = 0.82, respectively. Besides analyzing the scores dimensionally, Ruch et al. (2014) suggested identifying class clowns by averaging the items 4 and 9 (i.e., “My classmates would call me a class clown.” and “In my class I am the class clown.”), and consider those to be class clowns that had scores between 4 (partially agree) and 6 (totally agree). In the present sample the two items correlated highly, *r* = 0.84 (*p* < 0.001), and 18.3% of the participants had scores reaching the “partially agree” cut-off point for being classified as a class clown. In the following analyses, this score was used as *class clown status index*, with values below “partially agree” indicating not being considered a class clown and values above the cut-off point indicating being considered a class clown. Those considered class clowns were 29.4% of the boys and 8.3% of the girls.

For the teacher ratings of class clown behavior, four items consisting of descriptions of each of the four dimensions of class clown behavior as assessed by the CCBS were provided. Classroom teachers rated the extent to which they agreed that these items described a student’s typical behavior in the classroom on a 6-point scale (1 = *totally disagree* to 6 = *totally agree*). The total score across the four items yielded an internal consistency of α = 0.81. Similar to the self-reports, a class clown status index was computed for the teacher ratings using the item relating to class clown role (“The student would consider him/herself a class clown and is also referred to as class clown by his/her classmates”). Those students who received ratings of 4 (partially agree) or higher were considered class clowns (*n* = 58, i.e., 19.3% of the participants) in the analyses using the teacher ratings.

#### Peer Acceptance and Number of Friends

To assess peer acceptance, students were presented with a list of all their classmates and were asked to select all classmates they liked. The instructions read: “Please select those classmates that you like. These could be those that you like spending your breaks with or that you enjoy sitting next to.” They were also instructed that they could choose not to nominate any of their classmates. To determine a students’ peer acceptance, the number of received nominations was then adjusted for classroom size (i.e., divided by the number of participating students in the classroom minus 1). Consequently, the index for peer acceptance could range from 0 to 1. To determine the number of mutual friends in the classroom, students were asked to nominate their peers in the classroom whom they considered their friends by selecting them from a list of all students in the class, with a maximum of five nominations. A friendship was considered to be mutual when both friends had nominated each other. Thus, the number of mutual friends could range between 0 and 5.

#### Social Functioning in the Classroom

The *Revised Class Play* (Masten et al., 1985) was used to assess social behavior in the classroom. Students were presented with short behavior descriptions (e.g., someone who helps others) and were asked to nominate those students in their class that would be best suited to play this role in a hypothetical play. They did not have to nominate anyone and could nominate as many students as they wanted. To take different class sizes into account, the peer nominations a student received in each classroom were standardized. A German version of the instrument was developed using a standard translation-backtranslation procedure (Brislin, 1970). Four scales were computed according to Zeller et al. (2003): Popular-leadership (10 items), aggressive-disruptive (4 item; due to a negative corrected item-total correlation, the item “teases others” was deleted), sensitive-isolated (6 items), and prosocial (3 items; due to negative corrected item-total-correlations, the items “waits turn” and “is trustworthy” were deleted), yielded internal consistency coefficients of α = 0.90, α = 0.85, α = 0.71, and α = 0.85, respectively. The dimension *popular-leadership* contains items referring to sociable behavior with peers (e.g., someone who makes new friends easily) as well as to leadership skills (e.g., someone everyone listens to). *Aggressive-disruptive* behavior includes items in relation to disruption in the peer group (e.g., someone who fights a lot) and aggression toward others (e.g., someone who picks on others). The dimension *sensitive-isolated* consists of items that relate to withdrawn behavior characterized by difficulty interacting with peers (e.g., someone who is often left out). Finally, the dimension *prosocial* includes items that describe good manners with peers (e.g., someone who helps others). In addition to these four dimensions, a scale called *peer-perceived social position* was also considered as an additional indicator of social position, as suggested by Gest et al. (2001). It includes the items “has many friends,” “everyone likes to be with,” “has trouble making friends” (reverse-scored), and “is often left out” (reverse-scored) and yielded an internal consistency of α = 0.78 in the present sample.

### Procedure

Data for this study were collected in a classroom setting using school computers to complete the questionnaires that were presented online. Trained research assistants oversaw the completion of the questionnaires. Questionnaires were presented in two blocks, one block contained the nomination procedure for the assessment of peer acceptance and number of friends and the second block contained the Revised Class Play and the CCBS. The two blocks were presented in a randomized order. Classroom teachers completed the ratings at the same time or shortly after the student data had been collected also using an online questionnaire. The data presented here were collected as a part of a larger project and overlap with the sample used in Wagner (2018), which covers different variables and research questions. In total, students took between two and three lessons (i.e., between 90 and 135 min), including breaks, to complete all questionnaires.

## Results

### Preliminary Analyses

In preliminary analyses, it was tested whether the variables of interest were related to sex and age. Table 1 shows the descriptive statistics, both for the total sample and separately for boys and girls, as well as the correlations with age. Table 1 also shows the results of independent samples *t*-tests to test whether boys and girls differed in the studied variables.

**Table 1:** Descriptive statistics and correlations with sex and age for study variables.

	Total sample			Boys		Girls		Comparison	
	*M*	*SD*	*r*_Age_	*M*	*SD*	*M*	*SD*	*t*	*d*
**Class Clown Behavior Survey (Self-report)**									
Total score	2.56	1.05	0.04	2.86	1.08	2.29	0.95	4.90^∗∗∗^	0.57
Class clown role	2.24	1.25	−0.04	2.65	1.35	1.87	1.02	5.61^∗∗∗^	0.69
Comic talent	3.46	1.29	0.10	3.74	1.26	3.21	1.27	3.68^∗∗∗^	0.43
Disruptive rule breaker	2.24	1.18	0.05	2.52	1.26	1.98	1.06	3.95^∗∗∗^	0.47
Subversive joker	2.12	1.04	−0.03	2.37	1.10	1.90	0.93	4.04^∗∗∗^	0.47
**Class Clown Teacher Rating**									
Total score	2.05	1.08	0.07	2.35	1.17	1.78	0.91	4.72^∗∗∗^	0.58
Class clown role	1.97	1.41	0.07	2.40	1.59	1.59	1.09	5.10^∗∗∗^	0.65
Comic talent	2.72	1.54	0.08	3.08	1.67	2.39	1.34	3.91^∗∗∗^	0.47
Disruptive rule breaker	1.76	1.18	0.07	1.91	1.22	1.62	1.13	2.10^∗^	0.24
Subversive joker	1.75	1.20	0.01	2.01	1.35	1.50	1.00	3.70^∗∗∗^	0.46
**Nomination procedure**									
Peer acceptance	0.37	0.15	−0.16^∗∗^	0.37	0.15	0.37	0.15	0.13	0.02
Mutual friends	2.83	1.44	0.02	2.92	1.51	2.75	1.36	1.00	0.12

*N = 296–300 (total sample). Boys: n = 141–143. Girls: n = 155–157. ^∗^ p < 0.05, ^∗∗^ p < 0.01, and ^∗∗∗^ p < 0.001.*

In line with previous findings (Ruch et al., 2014), boys had higher scores on all of the four self-rated class clown dimensions, with effect sizes ranging between medium and large effects (see Table 1). This result was also found for the teacher ratings. Participants’ age was negatively related to peer acceptance, i.e., younger students were nominated more frequently as being liked by their classmates. As a consequence of these differences, sex and age were controlled for in the main analyses.

The teacher ratings of class clown behavior converged moderately with the self-reported CCBS scales (ρ = 0.42, ρ = 0.42, ρ = 0.36, and ρ = 0.35; all *p* < 0.001). The mean score across the four ratings correlated highly with the CCBS total score (ρ = 0.53; *p* < 0.001). The dimension *popular-leadership* was positively related to peer acceptance, *r* (296) = 0.68, and to the number of mutual friends, *r* (292) = 0.46, both *p* < 0.001, when controlling for influences of sex and age. The dimensions *aggressive-disruptive* and *sensitive-isolated* were negatively related to peer acceptance, *r* (296) = –0.19 and *r* (296) = –0.52, both *p* < 0.001, and the number of mutual friends, *aggressive-disruptive*: *r* (292) = –0.15, *p* = 0.009, *sensitive-isolated: r* (290) = –0.35, *p* < 0.001. The dimension *prosocial* was positively related to both aspects, *r* (296) = 0.30, *p* < 0.001, and *r* (292) = 0.17, *p* = 0.003 (all correlations controlled for sex and age). These findings replicate the results reported by Zeller et al. (2003), supporting the validity of the version of the measure used in the present study.

### Relationships of Class Clown Behavior With Social Status

Since the data had a nested structure (students nested in classrooms and schools), the lme4 package (Bates et al., 2015) in R (R Core Team, 2013) was used to compute multilevel random coefficients models. We tested the expected relationships in three-level random-intercept models, which means that a different intercept was estimated for every classroom (within every school). The standardized coefficients for the four class clown behavior scales (both in self-and teacher-ratings) predicting the different indicators of social status in the classroom (peer acceptance, number of mutual friends, and peer-perceived social position) while controlling for sex and age are displayed in Table 2.

**Table 2:** Results of multilevel models predicting peer acceptance, number of mutual friends, and peer-perceived social status (controlled for age and sex): Standardized coefficients for the fixed effects of the predictors (Class Clown Behavior Survey and Class Clown Teacher Rating) of the respective random-intercept models for each predictor entered separately.

	Peer Acceptance	Number of Mutual Friends	Peer-Perceived Social Status
**Class Clown Behavior Survey (Self-report)**			
Total score	0.22^∗∗∗^	0.16^∗∗^	0.36^∗∗∗^
Class clown role	0.20^∗∗∗^	0.18^∗∗^	0.34^∗∗∗^
Comic talent	0.22^∗∗∗^	0.17^∗∗^	0.36^∗∗∗^
Disruptive rule breaker	0.17^∗∗^	0.11	0.26^∗∗∗^
Subversive joker	0.18^∗∗^	0.09	0.030^∗∗∗^
Class clown status index (no = 0/yes = 1)	0.13^∗^	0.15^∗^	0.18^∗∗∗^
**Class Clown Teacher Rating**			
Total score	0.17^∗∗^	0.16^∗∗^	0.34^∗∗∗^
Class clown role	0.10	0.08	0.23^∗∗∗^
Comic talent	0.26^∗∗∗^	0.23^∗∗∗^	0.36^∗∗∗^
Disruptive rule breaker	0.06	0.05	0.23^∗∗∗^
Subversive joker	0.08	0.12	0.23^∗∗∗^
Class clown status index (no = 0/yes = 1)	0.05	0.04	0.16^∗∗^

*N = 296–300. ^∗^ p < 0.05, ^∗∗^ p < 0.01, and ^∗∗∗^ p < 0.001.*

As shown in Table 2, both the total score of the self-rating of class clown behavior and the total score of the teacher rating of class clown behavior were positively associated with all three indicators of social status (all *p* < 0.05). On the level of subscales, peer acceptance was positively related with all self-rated class clown behavior dimensions and with teacher-rated comic talent. The number of mutual friends was positively correlated with the two self-rated subscales of *identified as a class clown* and *comic talent* as well as the teacher-rated *comic talent* and *subversive joker*. Peer-perceived social position yielded positive correlations across all class clown behavior dimensions in both self- and teacher ratings.

However, the associations found with class clown status index (see Table 2) suggest that some of the relevant information is already explained by the fact whether someone sees him- or herself as a class clown or not. Class clowns tended to have higher peer acceptance, a higher number of mutual friends, and a higher peer-perceived social status than those that do not identify as class clowns. To determine the unique contribution of each class clown behavior dimension above the binary variable class clown status index and the other dimensions all predictors were entered simultaneously (separately for self-ratings and teacher-ratings of class clown behavior). As predictors, the covariates sex and age, the class clown status index (0 = not considered a class clown, 1 = considered a class clown) and the dimensions of class clown behavior were entered. The results of these analyses are presented in Table 3.

**Table 3:** Results of multilevel models predicting peer acceptance, number of mutual friends, and peer-perceived social status (controlled for age and sex): Standardized coefficients for the fixed effects of the predictors of the respective random-intercept models for each block of predictors (self- and teacher-ratings of class clown behavior) entered simultaneously.

	Peer Acceptance	Number of Mutual Friends	Peer-Perceived Social Status
**Class Clown Behavior Survey (Self-report)**			
Class clown status index (no = 0/yes = 1)	0.05	0.11	0.04
Comic talent	0.17^∗^	0.18^∗^	0.28^∗∗∗^
Disruptive rule breaker	0.00	0.00	−0.02
Subversive joker	0.05	−0.07	0.11
**Class Clown Teacher Rating**			
Class clown status index (no = 0/ yes = 1)	−0.10	−0.09	−0.09
Comic talent	0.30^∗∗∗^	0.25^∗∗^	0.34^∗∗∗^
Disruptive rule breaker	0.00	−0.07	0.10
Subversive joker	0.01	0.11	0.07

*N = 296–300. ^∗^ p < 0.05, ^∗∗^ p < 0.01, and ^∗∗∗^ p < 0.001.*

To avoid problems with multicollinearity, the self-reported dimension of class clown role was excluded from these analyses since two of the four items forming the scale were also used to build the class clown status index resulting in a high correlation between the dimension class clown role and the class clown status index, *r*(300) = 0.76. As shown in Table 3, only the dimension of comic talent uniquely predicted peer acceptance, number of mutual friends and peer-perceived social position. The class clown status index as well as the other dimensions of class clown behavior did not show any significant relationships with the outcomes in these analyses.

It was also tested whether the presented relationships were moderated by sex by including an interaction term between the class clown dimension and sex as additional predictor. No moderation effects were observed for any of the combinations of class clown dimensions and indicators of social status (all *p* > 0.05).

### Relationships of Class Clown Behavior With Social Functioning in the Classroom

Table 4 shows the standardized coefficients for the fixed effects in the random-intercept models predicting the four dimensions of social behavior in the classroom (popular-leadership, aggressive-disruptive, sensitive-isolated, and prosocial) as nominated by peers from self- and teacher-reported class clown behavior.

**Table 4:** Results of multilevel models predicting dimensions of classroom behavior, as assessed by the revised class play (controlled for age and sex): Standardized coefficients for the fixed effects of the predictors (Class Clown Behavior Survey and Class Clown Teacher Rating) of the respective random-intercept models for each predictor entered separately.

	Popular-leadership	Aggressive-disruptive	Sensitive-isolated	Prosocial
**Class Clown Behavior Survey (Self-report)**				
Total score	0.23^∗∗∗^	0.26^∗∗∗^	−0.09	−0.27^∗∗∗^
Class clown role	0.22^∗∗∗^	0.26^∗∗∗^	−0.04	−0.26^∗∗∗^
Comic talent	0.23^∗∗∗^	0.24^∗∗∗^	−0.09	−0.21^∗∗∗^
Disruptive rule breaker	0.16^∗∗^	0.22^∗∗∗^	−0.06	−0.26^∗∗∗^
Subversive joker	0.17^∗∗^	0.17^∗∗^	−0.10	−0.21^∗∗∗^
Class clown status index (no = 0/ yes = 1)	0.16^∗∗^	0.27^∗∗∗^	0.05	−0.21^∗∗∗^
**Class Clown Teacher Rating**				
Total score	0.23^∗∗∗^	0.42^∗∗∗^	0.05	−0.39^∗∗∗^
Class clown role	0.11	0.36^∗∗∗^	0.08	−0.30^∗∗∗^
Comic talent	0.25^∗∗∗^	0.28^∗∗∗^	−0.06	−0.21^∗∗∗^
Disruptive rule breaker	0.04	0.36^∗∗∗^	0.09	−0.42^∗∗∗^
Subversive joker	0.14^∗^	0.33^∗∗∗^	0.08	−0.36^∗∗∗^
Class clown status index (no = 0/yes = 1)	0.07	0.31^∗∗∗^	0.09	−0.26^∗∗∗^

*N = 300. ^∗^ p < 0.05, ^∗∗^ p < 0.01, and ^∗∗∗^ p < 0.001.*

The pattern of associations displayed in Table 4 shows that, the class clown behavior *total score* was positively related to the scales *popular-leadership* and *aggressive*. It was unrelated to the scale *sensitive-isolated* and negatively related to *prosocial*. The class clown behavior dimensions *comic talent* and *subversive joker* showed consistent positive correlations to *popular-leadership* across both self- and teacher ratings. *Aggressive-disruptive* classroom behavior was consistently related to all dimensions of class clown behavior. The *sensitive-isolated* scale was unrelated to the class clown behavior dimensions. There were medium-sized negative correlations between all class clown dimensions and *prosocial* classroom behavior.

Again, the *class clown status index* seemed to carry some of the relevant variance. Considering oneself to be a class clown yielded higher scores in peer-rated popular and aggressive classroom behavior and lower scores in prosocial behavior. To determine the unique contribution of each class clown behavior dimension above the binary variable class clown status index and the other dimensions, all predictors were entered simultaneously (excluding the dimension of self-rated class clown role) for each of the classroom behavior dimensions. The results of these analyses are presented in Table 5.

**Table 5:** Results of multilevel models predicting dimensions of classroom behavior, as assessed by the revised class play (controlled for age and sex): Standardized coefficients for the fixed effects of the predictors of the respective random-intercept models for each block of predictors (self- and teacher-ratings of class clown behavior) entered simultaneously.

	Popular-leadership	Aggressive-disruptive	Sensitive-isolated	Prosocial
**Class Clown Behavior Survey (Self-report)**				
Class clown status index (no = 0/yes = 1)	0.08	0.21^∗∗^	0.12	−0.10
Comic talent	0.21^∗^	0.17^∗^	−0.07	−0.07
Disruptive rule breaker	−0.02	0.09	0.03	−0.20^∗^
Subversive joker	0.02	−0.10	−0.12	0.04
**Class Clown Teacher Rating**				
Class clown status index (no = 0/yes = 1)	−0.06	0.11	0.12	−0.04
Comic talent	0.27^∗∗∗^	0.11	−0.16^∗^	−0.02
Disruptive rule breaker	−0.13	0.17^∗^	0.06	−0.31^∗∗∗^
Subversive joker	0.15	0.13	0.05	0.13

*N = 300. ^∗^ p < 0.05, ^∗∗^ p < 0.01, and ^∗∗∗^ p < 0.001.*

Table 5 shows that when considering self-reported class clown behavior, comic talent uniquely predicted the dimension of *popular-leadership*. *Aggressive-disruptive* was predicted by both the class clown status index and comic talent. *Sensitive-isolated* was not predicted by class clown behavior, and *prosocial* was uniquely negatively related to the dimension of disruptive rule-breaker. When considering teacher-rated class clown behavior, comic talent uniquely predicted *popular-leadership*. Class clown status index and disruptive rule-breaker both predicted *aggressive-disruptive* behavior. *Sensitive-isolated* was uniquely negatively related with comic talent, and *prosocial* was negatively related with both class clown status index and disruptive rule-breaker.

To get a clearer picture of the relationships of class clown behavior with aggressive-disruptive classroom behavior, an additional exploratory analysis was performed. In this analysis, the extent to which the class clown status index and the class clown behavior dimensions predicted the individual items of the RCP scale was inspected by multilevel models parallel to those performed on the full scale. These analyses showed that using the self-rated class clown behavior dimensions, all items were predicted by the class clown status index (all *p* < 0.05). When using the teacher-rated class clown behavior dimensions, all items were predicted by the class clown status index and the dimension of disruptive rule-breaker (all *p* < 0.05). However, the item “too bossy” was additionally predicted by the dimension of comic talent in both self- and teacher ratings (*p* < 0.05).

It was also tested whether these relationships were moderated by sex by including an interaction term between the class clown dimension and sex as predictors for all four dimensions of classroom behavior as criteria. Overall, only two combinations of class clown dimensions and classroom behavior dimensions yielded a moderating effect of sex (*p* < 0.05); the positive relationship between *comic talent* and aggressive-disruptive behavior was stronger for boys than for girls and the positive relationship between *disruptive rule-breaker* and popular-leadership was stronger for girls than for boys (all analyses controlling for age).

## Discussion

The present study used different data sources (self-reports, peer nominations, and teacher ratings) to investigate how different dimensions of class clown behavior relate to the social status and social functioning of the students habitually displaying such behaviors. Overall, the results underline the relevance of class clown behavior for social functioning in the classroom. Like in prior studies (Ruch et al., 2014; Platt et al., 2016), the different dimensions of class clown behaviors (in particular comic talent and aggressive-disruptive) differentially affected outcome measures.

With regard to the first hypothesis, as expected, class clown behavior both from the perspectives of the students and the teachers generally went along with higher social status, that is being well-liked and having many friends as well as being perceived as well-liked and having many friends by one’s classmates. When considering the overlap between the different dimensions of class clown behavior, *comic talent* was the most relevant one carrying the strongest associations with social status. Those who like to entertain their classmates with funny things and are quick-witted are well-accepted in the classroom, have many friends, and have a reputation for being well-liked and having many friends. As expected in the second hypothesis, class clown behavior was also related to higher scores on the dimensions popular-leadership. Again, the dimension of *comic talent* uniquely predicted the classroom behavior dimension of popular-leadership when considering the overlap between the dimensions and thus seems to be most relevant when predicting this behavior. Those who express humor in the classroom by sharing funny things with their classmates also tend to be considered leaders in the classroom.

Also the third and fourth hypotheses were confirmed, namely class clown behavior was associated with higher scores on the classroom behavior dimension of aggressive-disruptive, and lower scores on the dimension prosocial. As predicted, the dimension of *disruptive rule-breaker* was most relevant when explaining differences in aggressive-disruptive behavior (for teacher-rated class clown behavior) and in (low) prosocial behavior. Those students who like to mock the teachers and to poke fun at the school rules are characterized as showing aggression in the classroom and as not being polite and helpful (cf. Platt et al., 2016). These findings are corroborated by the generally high convergence between the analyses using self- and those using teacher-rated class clown behavior.

Taken the results concerning the four hypotheses together, class clown behavior went along with positive (higher scores on popular-leadership), the absence of positive (lower scores on prosocial), and negative (higher scores on aggressive-disruptive) aspects of social behavior in the classroom. In light of the large amount of research showing links between social status and different forms of aggression (for a review, see e.g., Heilbron and Prinstein, 2008), this co-occurrence is not surprising. It might be interesting to look at class clown behavior in more detail as an example of a type of behavior that seems to contribute to both social status and aggressive behavior in the classroom.

Exploratory analyses were conducted on each of the items of the aggressive-disruptive scale of the RCP to generate ideas for a more detailed understanding of the relationships between the different dimensions of class clown behavior and the display of aggressive behavior in the classroom. These results suggest that while the class clown status is related with aggressive-disruptive behavior in general, the dimensions predicted the items differentially and different kinds of class clown behavior seem to involve different kinds of aggressive behaviors – the comic talents are perceived as dominant and self-opinionated (“too bossy”) and the disruptive rule-breakers are perceived as verbally and/or physically aggressive (“picks on others” and “gets into fights”). In future studies, it might be worthwhile to look at different forms of aggressive behavior in more detail to gain a deeper understanding of these relationships.

The current results clearly support the usefulness of a dimensional approach when compared to a typological approach in studying class clown behavior. Within the dimensional approach, it seems that the dimensions of comic talent and disruptive rule-breaker showed clearly different patterns of associations, whereas the dimension of subversive joker did not emerge as unique predictor of any of the variables in the present study. Future research will be needed to critically examine whether it can predict other variables beyond the other dimensions. When comparing the two approaches, the dichotomous variable *class clown status index*, which categorized students into “class clowns” and “not-class clowns,” also showed relations with the studied variables. This finding underlines that it matters whether or not a student perceives him- or herself as a class clown. However, when entered together with the dimensions of class clown behavior, the dimensions – in particular *comic talent* and *disruptive rule-breaker* – mostly outperformed the class clown status index in predicting the outcomes of interest. This shows that while the label “class clown” does have some relevance, the more powerful distinction is which kind of class clown behavior a student shows. For future research, it seems to be promising to move beyond studying “class clowns” as compared to “not-class clowns,” which also requires somewhat arbitrary cut-offs, and to consider class clown behavior as a dimensional *and* multidimensional phenomenon.

With respect to sex differences, the present study replicates previous findings regarding the higher prevalence of class clown behavior among boys compared to girls. Regarding our substantive research questions, there was little evidence of moderating effects of sex in the studied relationships. The very few sex differences are nonetheless in line with previous findings (e.g., Barnett, 2018): Boys showed stronger associations of class clown behavior with negative outcomes and weaker associations with positive outcomes than girls did. This might be due to teachers and peers perceiving humorous behavior in the classroom more negatively in boys than in girls as suggested by Barnett (2018). It has to be noted though that a comparison to the findings by Barnett (2018) is hampered by the use of different age groups. The sample in Barnett’s study was on average 9 years old at the last data collection, while the present sample consisted of adolescents who were on average 13 years old. It can be assumed that class clown behavior itself, as well as its perception by teachers and peers and its correlates, changes with age and also by the type of school a student attends. Studies using large samples from different age groups as well as additional longitudinal studies are needed to enhance our understanding of these processes.

Similarly, the perception of classroom behavior and its consequences seems to vary depending on the perspective (self, teacher, or peer). In the present study, self- and teacher-ratings of class clown behavior were considered. They converged moderately and also showed a generally similar pattern of results, even though the convergence was not perfect. Several reasons for this are conceivable. First, some of the behaviors are addressed toward the peers and might thus be less visible for the teacher. Second, in particular in the case of the class clown behavior dimension of subversive joker, students poke fun at the teachers behind their backs – so it should be more difficult for them to observe the behavior. Third, even though teachers in the present study were teaching students for a significant amount of lessons in a week, there were in most cases also other teachers who were teaching in the respective classroom, so one teacher would not be able to observe behavior in all lessons and with all teachers. In future studies, it would be interesting to also assess peer ratings on class clown behavior. In general, it seemed that in the teacher ratings the distinction between “positive” (comic talent) and “negative” class clown behavior was amplified. For instance, teacher-rating on the dimension *disruptive rule-breaker* were not related to peer acceptance or *popular-leadership* classroom behavior, while there were positive relationships with self-ratings of this dimension. Taken together with the observation that the means of the teacher ratings were generally lower than those of the self-ratings, it might be the case that teachers had a higher threshold for noticing or describing class clown behaviors, in particular disruptive ones, and thus their ratings might be more sensitive for more extreme behaviors. Future research might benefit systematically comparing self-, teacher- and peer-reports of class clown behavior, but also from extending beyond those perspectives. One approach could be observing distinct behaviors perceived as class clown behavior instead of generalized dimensions. The study of such distinct behaviors might lead to a clearer understanding what is perceived as class clown behavior, how different behaviors are appreciated, and how classmates and teachers react to different kinds of class clown behavior.

When interpreting the present results, it might also be useful to consider the different profiles of character strengths that have been found to be associated with the different dimensions of class clown behavior (Ruch et al., 2014). Character strengths as described in the VIA classification (Peterson and Seligman, 2004) represent a family of positive traits that can contribute to a “good life,” and with that, they are related to a number of positive outcomes, including positive relationships. Recently, Wagner (2018) identified a number of character strengths as most relevant to adolescents’ peer relationships and friendships in the classroom. The class clown behavior dimension of comic talent was found to be associated with a number of character strengths that overlap with those identified as most relevant for peer relationships; most notably perspective, love, social intelligence, leadership, and (naturally) humor (Ruch et al., 2014). While Ruch et al. (2014) found that the other three dimensions of class clown behavior were also associated with the character strength of humor (though to a lesser degree), they were not associated with most of the strengths mentioned and even displayed some negative correlations with strengths that have been identified as instrumental for social functioning in the classroom, such as honesty or teamwork. Future research might also aim to understand which additional individual differences underlie the different dimensions of class clown behavior. For instance, does being a “comic talent” and showing quick-witted humor behavior in fact go along with high (verbal) intelligence (cf. Masten, 1986)?

Some limitations of the present study need mentioning. Firstly, the reported results are cross-sectional associations, not allowing for any conclusions regarding directionality or causality. Secondly, the measure used to assess teacher perceptions of class clown behavior was based on single items, limiting the reliability of the assessment. Thirdly, the psychometric properties of the Revised Class Play scales were not consistently desirable. The German translation used has not been validated previously, and thus these results need to be interpreted with some caution. Finally, while there is initial evidence on the validity of the CCBS (Ruch et al., 2014; Platt et al., 2016), further work is needed to corroborate its validity and to also test it systematically in different age groups.

## Conclusion

The present study underlines that displaying humor in the classroom in the form of class clown behavior has both upsides add downsides for the individual. In general, humor is related not only to a life of pleasure, but also to an orientation to positive relationships (Wagner et al., 2019). Our results show that this is also true for class clown behavior – in particular one kind of class clown behavior, the comic talent. This dimension was found to be uniquely related with being well accepted by one’s classmates and having many friends in the classroom, which are important aspects of positive peer relationships in adolescence. Teachers confronted with the expression of humor in the classroom might thus benefit from focusing on its positive effects (i.e., on relationships in the classroom and on the atmosphere, see also Meeus and Mahieu, 2009). There are, however, also clear downsides to class clown behavior with respect to social functioning in the classroom, in the form of aggressive behavior or low prosocial behavior.

The present results support a dimensional approach looking beyond assuming versus not assuming the role of a class clown. While the dimension of disruptive rule-breaker is related to various downsides and the dimension of comic talent seems to have many upsides when it comes to social functioning in the classroom, comic talent also went along with more aggressive behavior. Class clown behavior and its relationship with social functioning in the classroom, it seems, is not a black and white issue.

## Ethics Statement

This study was carried out in accordance with the recommendations of the Swiss Psychological Association and with the Declaration of Helsinki. All participants provided written informed consent. In line with local guidelines, participants under the age of 14 years also provided the written informed consent of a parent or legal guardian. The local ethics committee at the University of Zurich also approved the procedures (including the consent procedures) before the start of the study.

## Author Contributions

LW designed the study, supervised data collection, conducted the data analysis, and wrote the manuscript.

## Conflict of Interest Statement

The author declares that the research was conducted in the absence of any commercial or financial relationships that could be construed as a potential conflict of interest.
